# Retraction: MicroRNA-497 inhibits thyroid cancer tumor growth and invasion by suppressing BDNF

**DOI:** 10.18632/oncotarget.28248

**Published:** 2022-06-30

**Authors:** Peisong Wang, Xianying Meng, Yan Huang, Zhi Lv, Jia Liu, Guimin Wang, Wei Meng, Shuai Xue, Qiang Zhang, Pengju Zhang, Guang Chen

**Affiliations:** ^1^Department of Thyroid Surgery, The First Hospital of Jilin University, Chaoyang District, Changchun 130021, China; ^2^Department of Orthopaedic Surgery, China-Japan Union Hospital of Jilin University, Jingyue District, Changchun 130033, China; ^3^Jilin Academy of Agricultural Sciences, Nanguan District, Changchun 130000, China; ^*^These authors have contributed equally to this work

**This article has been retracted:** Oncotarget has completed its investigation of this paper. We found that Figures 2C, 2E, 6B, and 6F contain similarities with data appearing in other articles by different authors. The data had already been published elsewhere, or were already under consideration for publication prior to submission to Oncotarget. On June 15, 2022 Oncotarget received a signed letter asking to retract the paper because certain experiments, including cell colony formation and *in vivo* assay, were unable to be reproduced. Oncotarget has notified the affiliated institution regarding this retraction.

Original article: Oncotarget. 2017; 8:2825–2834. 2825-2834. https://doi.org/10.18632/oncotarget.13747

